# Morphology and molecular characterization of *Ceratomyxa* sp. (Cnidaria, Myxosporea, Ceratomyxidae), infecting the gallbladder of *Curimata cyprinoides* (Characiformes: Curimatidae) in the lower Araguaia River, Brazil

**DOI:** 10.1590/S1984-29612025014

**Published:** 2025-05-23

**Authors:** Maria Josinete Araujo Costa, Maria Queroz Carneiro Silva, Marcelo Francisco da Silva, Evonnildo Costa Gonçalves

**Affiliations:** 1 Programa de Pós-graduação em Biologia de Agentes Infecciosos e Parasitários – BAIP, Universidade Federal do Pará – UFPA, Belém, PA, Brasil; 2 Laboratório de Ecologia e Limnologia – LEL, Universidade Estadual da Região Tocantina do Maranhão – UEMASUL, Imperatriz, MA, Brasil

**Keywords:** Gallbladder parasite, SSU Rdna, fish parasite, Araguaia river, eastern Amazon region, Parasita da vesícula biliar, ADNr de SSU, parasito de peixe, rio Araguaia, região leste da Amazônia

## Abstract

This study reports the occurrence of a myxosporid parasite of the genus *Ceratomyxa* Thélohan, 1892 found in the gallbladder of *Curimata cyprinoides* Linnaeus, 1766. This species is abundant in the Tocantins-Araguaia hydrographic basin and holds environmental, social and economic importance. The genus *Ceratomyxa* is characterized by two equally-sized polar capsules with lateral projections, whick may appear slightly slightly arched in a half-moon shape or fully curved arched. Light microscopy and molecular analysis were employed in this study. The mature spores were composed of two equally-sized symmetrical valves with equal capsular foramina and two equally-sized lateral projections. These spores (n=40) were freely suspended in the gallbladder, with a total length of 11.2 ± 0.1 μm and width of 4.0 ± 0.3 μm. The polar capsules measured 2.1 ± 0.1 μm in both length and width; and the two symmetrical lateral elongations formed a posterior angle of 41.4º ± 0.7º. An integrated comparative analysis of the morphological characteristics and partial SSU rDNA sequences confirmed this finding as a parasite of the genus *Ceratomyxa* sp., located in the gallbladder of *C. cyprinoides* in the Tocantins-Araguaia basin, within the municipality of Araguatins, in eastern Amazon, Brazil.

## Resumo

Este estudo relata a ocorrência de um parasita mixosporídeo do gênero *Ceratomyxa* Thélohan, 1892, encontrado na vesícula biliar de *Curimata cyprinoides* Linnaeus, 1766. Essa espécie é abundante na bacia hidrográfica Tocantins-Araguaia e possui importância ambiental, social e econômica. O gênero *Ceratomyxa* é caracterizado por duas cápsulas polares de tamanhos iguais com projeções laterais, que podem parecer ligeiramente arqueadas em forma de meia-lua ou totalmente curvadas. Microscopia óptica e análise molecular foram empregadas neste estudo. Os esporos maduros eram compostos por duas válvulas simétricas de tamanhos iguais, com forames capsulares iguais e duas projeções laterais de tamanhos iguais. Esses esporos (n=40) ficaram suspensos livremente na vesícula biliar, com comprimento total de 11,2 ± 0,1 μm e largura de 4,0 ± 0,3 μm. As cápsulas polares mediam 2,1 ± 0,1 μm em comprimento e largura; e os dois alongamentos laterais simétricos formaram um ângulo posterior de 41,4º ± 0,7º. Uma análise comparativa integrada das características morfológicas e sequências parciais de rDNA do SSU confirmou esse achado como um parasita do gênero *Ceratomyxa* sp., localizado na vesícula biliar de *C. cyprinoides* na bacia Tocantins-Araguaia, no município de Araguatins, no leste da Amazônia, Brasil.

Members of the phylum Myxozoa Grassé, 1970, are among the most common parasites of aquatic organisms, affecting both vertebrates and invertebrates across marine and freshwater environments (Okamura et al., 2015; Thabet et al., 2016). Within this phylum, the family Ceratomyxidae Doflein, 1899, includes the genera *Ceratomyxa* Thélohan, 1892, and *Leptotheca* Thélohan, 1895. Species within Ceratomyxidae are myxosporidian parasites characterized by elongated spores that are half-moon-shaped or arched, with often conical shells, resulting in a spore length greater than its diameter (Lom & Dyková, 2006).

In this study, parasites were found inside vermiform plasmodia in the gallbladder of the characiform fish *Curimata cyprinoides* Linnaeus, 1776 based on morphological characteristics, it is inferred that this parasite belongs to specie within genus *Ceratomyxa* for which some, including *C. mandii* (Araújo et al., 2022), *C. amazonensis* (Mathews et al., 2016), *C. vermiformis* (Adriano & Okamura, 2017), *C. brasiliensis* (Zatti et al., 2017), *C. gracillima* (Zatti et al., 2017) *C. fonsecai* (Silva et al., 2020) and, most recently, *C. tartarugalis* (Araújo, 2021), have been previously reported in the Amazon region, with their identification corroborated by molecular techniques.

In the Amazon region, fish in the family Curimatidae, such as *C. cyprinoides,* known as “branquinha”, are crucial for riverbank and commercial subsistence (Santos et al., 2006; Soares et al., 2011) native to South America, this species is distributed across the Orinoco River delta the Atlantic drainage area of the Guianas, and the lower Amazon and Tocantins Rivers. Despite the economic, ecological, and environmental significance of these fish, their parasitology and the potentian health implications of their parasites remain underexplored.

This study aimed to describe a *Ceratomyxa* species through light microscopy and phylogenetic analysis, identifying parasite in the gallbladder of *C. cyprinoides* specimens.

Thirty-three fish were sampled from the Araguaia River, near Araguatins (5.646035 ºS / 48.131194 ºW), in Tocantins, Brazil under the authorization of the Brazilian Ministry of the Environment (MMA/SISBIO 75916-4). The fish were kept alive, transported, and maintained in aquariums for up to 12 hours at in the Ecology and Limnology Laboratory (LEL), State University of the Tocantina Region of Maranhão (UEMASUL), in Imperatriz, MA, Brazil. They were anesthetized with tricaine methanesulfonate (MS222; SIGMA) at 50 mg/L weighed (in grams) measured (cm), and dissected for parasites detection.

This analysis was conducted with the approval of the UEMASUL ethics Committee for Experimental Animal Use (CEUA/UEMASUL authorization no. 6186201221) each fish`s specimen, the fins, eyes, mouth, opercula, gills, and gastrointestinal tract were examined for parasites, with organ fragments further analyzed were removed and examined under a light microscope.

Photomicrographs of fresh spores were obtained using phase contrast on a Zeiss Axiovert A1 microscope equipped with an the AxionCam ICc1 and ZEN (blue edition) 2.3 software.

Fragments containing in which the presence of mature spores were analyzed morphometrically using the Axiovert A1 microscope. Measurements in micrometers (µm) were recorded for key the following morphometric parameters: spore body length (L), spore body thickness (T), posterior angle (θ), polar capsule length (CL); and polar capsule width (CW). These parameters were adapted from the methodologies of Lom & Arthur (1989) and Heiniger et al. (2008). Prevalence was estimated according to Bush et al. (1997).

For molecular and phylogenetic analyses, gallbladder samples infected with myxozoan spores were removed and preserved in and fixed in 80% ethanol. DNA extraction was conducted with the PureLink® genomic DNA mini-kit (Invitrogen, USA), following the manufacturer’s protocol. The extracted DNA was samples were quantified using Biodrop Duo spectrophotometer (Biodrop) and subsequently used in polymerase chain reaction (PCR) technique to obtain the partial sequence of small subunit ribosomal DNA (SSU rDNA), with using primers recommended in the literature. The ERIB1/ERIBI10 primer set (Barta et al., 1997) was used in the initial amplification round, followed by Myxogen4f and 18R MC3/MC5 primers (Whipps et al., 2003; Diamant et al., 2004) for the second round.

The final PCR reaction was prepared in a carried out with a reaction volume of 25 µl volume with using Taq DNA polymerase Master Mix (PROMEGA, Madison, USA), 0.5µl of each primer, and 2 µl of DNA template sample. PCR conditions, followed were adjusted in accordance protocols from described Whipps et al. (2003), with an initial denaturation . The amplification was started with denaturing at 94 °C for 30 seconds, followed by 36 cycles of denaturing at 94 °C for 30 seconds, annealing hybridization at 58 °C for 40 seconds, and extension polymerization at 72 °C for 45 seconds. A second amplification round included, initial denaturation at 94 °C for 2.5 minutes, followed by 30 cycles of denaturing at 94 °C for 20 seconds, annealing hybridization at 46 °C for 30 seconds, and extension polymerization at 65 °C for one minute, concluding with a and then final extension polymerization at 65 °C for 10 minutes. PCR products were visualized through agarose gel electrophoresis and purified with using the GFX^TM^ PCR DNA Purification Kit (GE Healthcare, United Kingdom). Partial SSU rDNA sequences were obtained using the Big Dye Terminator v3.1 sequencing kit (Applied Biosystems, Foster City, USA), per in the manufacturer’s recommendations.

Sequences were compiled in BioEdit (Hall, 1999) and compared with other ceratomyxid sequences CLUSTALW alignment was applied to identify homologous regions and eleminate non informative variable regions (Holzer et al., 2007; Gunter et al., 2009).

Phylogenetic analyses were conducted based on the general time-reversible (GTR + R) evolutionary model, selected using through jModeltest (Posada, 2008). Maximum likelihood (ML) analysis was performed with carried PAUP v.4.0a161 (Swofford, 2003), employing with 10,000 bootstrap replicates, while and Bayesian evolutionary analysis was conducted via the multiplatform BEAST v.1.8.4 software (Drummond & Rambaut, 2007), using with a relaxed, and non-correlated lognormal model based on the birth-death speciation process (Mooers et al., 2012). The with a main phylogenetic tree generated using the unweighted pair group method with arithmetic mean (UPGMA). Markov chain Monte Carlo (MCMC) simulation ran for performed for 10,000,000 with sampling every 10,000 passes. Convergence and tree assessment were carried our using Tracer (Rambaut et al., 2018), TreeAnnotator v1.8.4 (Drummond & Rambaut, 2007) and FigTree v.1.4.3, with 12% burn-in, applied to generate the maximum credibility clade tree and edit the final phylogenetic tree.

The *C. cyprinoides* specimens analyzed were collected from the eastern Amazon, in the lower Araguaia River near in the urban area of Araguatins (5.646035 °S / 48.131194 °W), Tocantins, Brazil. Their mean weight was 88.4 ± 14.8 g (52-119 g) and their mea mean total length was 17.6 ± 1.6 cm (14.5-21.0 cm). The sample consisted of 14 females and 19 males with no significant difference in infection rates between sexes. in relation to Seven of the 33 fish examined (21%) exhibited presented myxozoan spores, found either in plasmodia or freely dispersed within the gallbladder fluid.

**Figure 1:**
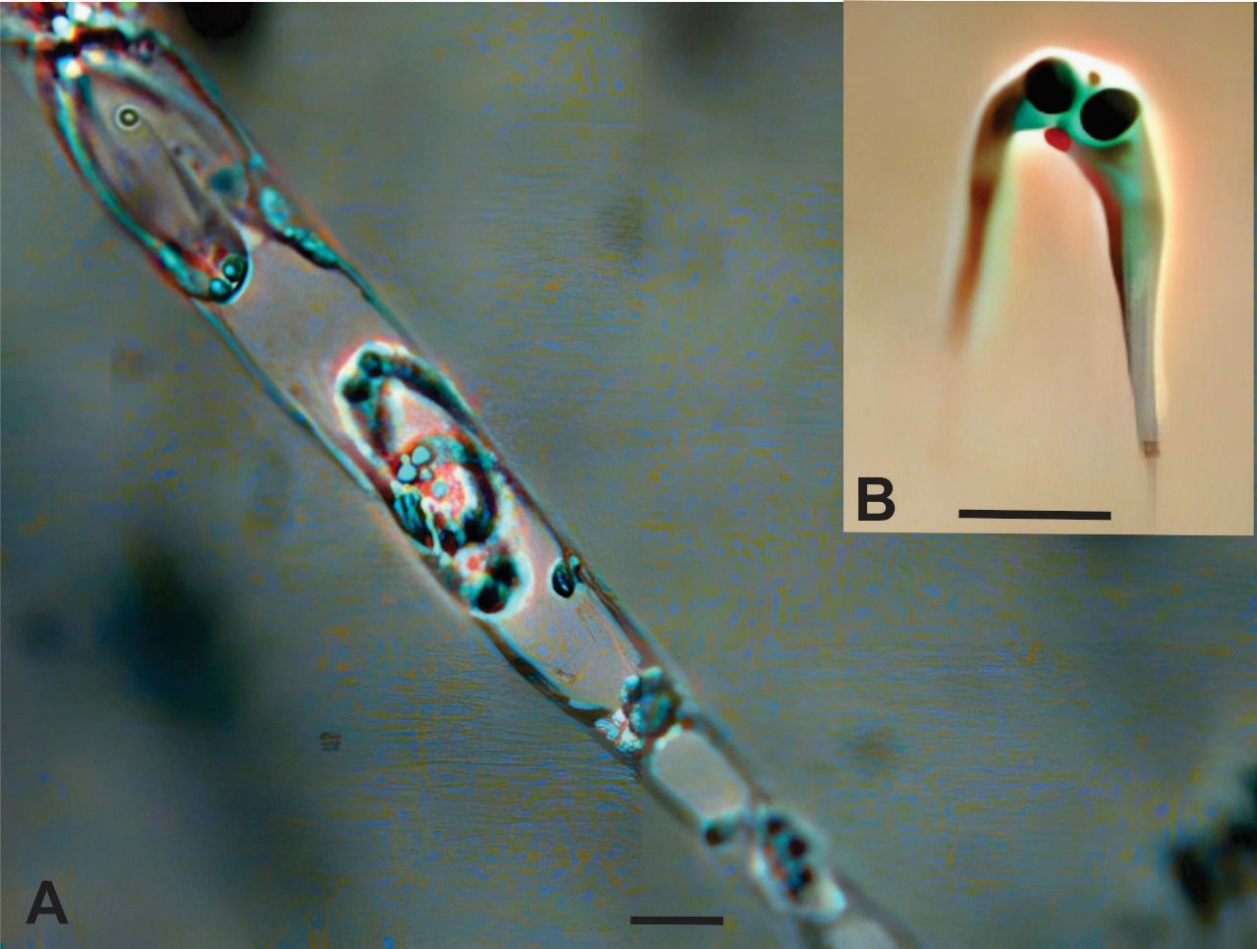
(A) Photomicrograph of mature plasmodia; (B) Mature spore of *Ceratomyxa* sp. Bars = 10 μm.

The vermiform plasmodia were polysporous, exhibiting presenting oscillatory movement, and were dispersed within the gallbladder. Immature plasmodia displayed amorphous shapes (Figure 1A).

Mature spores were found dispersed throughout the gallbladder contents, either individually singly or in groups, after release from ruptured plasmodial membranes plasmodium (Figure 1B). These mature myxospores (n=40) were highly strongly curved, measuring 11.2 ± 0.1 μm in length and 4 ± 0.3 μm in thickness, with a posterior angle of 41.4 ± 0.7 degrees. Within Inside the myxospores, there were two a pair subspherical polar capsules with equal capsular foramina, each measuring 2.01 ± 0.1 μm in length and 2.02 ± 0.1 μm in width.

**Figure 2:**
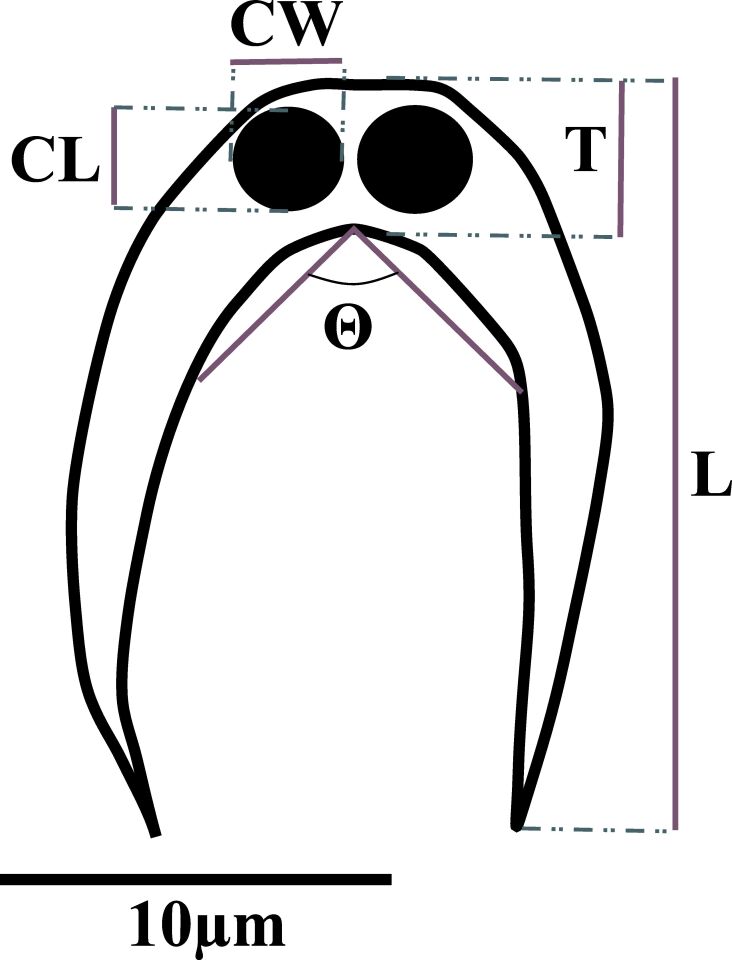
*Ceratomyxa* sp. mature spore illustration. L: spore length; T: spore thickness; CL: capsule length; CW: capsule width; θ: posterior angle.

Based on the observed of morphological characteristics (Figure 2), these myxospores display presented the typical pattern of seen in the genus *Ceratomyxa* Morphological differences observed among the *Ceratomyxa* specimens described in this study and other the species known described to as parasitize fish in the Amazon region are outlined detailed in Table 1.

**Table 1:** Morphometric data available in relation to species of Ceratomyxa in the Amazon region.

Species	Parameters									Reference
	L	T	T:L Ratio	CL	CW	Θ	Bob	Site	Host	
*Ceratomyxa* sp.	11.2 ± 0.1	4.0 ± 0.3	2.8	2.01 ± 0.1	2.02 ± 0.1	41.4 ± 0.7	*	Gallbladder	*Curimata cyprinoides*	This study
*C. tartarugalis*	31.95 ± 0.4	2.15 ± 0.06	14.86	1.7 ± 0.46	1.31 ± 0.06	*	*	Gallbladder	*Hemiodus gracilis*	Araujo (2021)
*C. gracillima*	4.4 ± 1.1	28.0 ± 3.4	6.36	1.9 ± 0.4	*	37 ± 2.9	2-3	Gallbladder	*Brachyplatystoma rousseauxii*	Zatti et al. (2017)
*C. amazonensis*	7.0 ± 0.3	15.8 ± 0.4	2.24	3.2 ± 0.3	3.6 ± 0.2	103.7 ± 10.3	3-4	Gallbladder	*Symphysodon discus*	Mathews et al. (2016)
*C. microlepis*	5.2 ± 0.4	35.5 ± 0.9	6.83	2.2 ± 0.3	-	162.3 ± 4.3	5-6	Gallbladder	*Hemiodus microlepis*	Azevedo et al. (2013)
*C. vermiformis*	4.5 ± 0.2	23.7 ± 0.7	5.24	2.7 ± 0.1	-	30.2 ± 6.6	3-4	Gallbladder	*Colossoma macropomum*	Adriano & Okamura (2017)
*C. mylei*	5.1 ± 0.3	24.6 ± 0.8	4.82	2.1 ± 0.3	-	37 ± 6	5-6	Gallbladder	*Myleus rubripinnis*	Azevedo et al. (2011)
*C. brasiliensis*	6.3 ± 0.6	41.2 ± 2.9	6.54	2.6 ± 0.3	2.5 ± 0.4	147 ± 5.1	3-4	Gallbladder	*Cichla monoculus*	Zatti et al. (2017)
*C. fonsecai*	2.6 ± 0.1	28.9 ± 2.7	11.12	1.9 ± 0.3	1.7 ± 0.2	164.8 ± 8.6	3-4	Gallbladder	*Hemiodus unimaculatus*	Silva et al. (2020)
*C. macapaensis*	4.2 ± 0.5	22.7 ± 0.3	5.41	1.86 ± 0.3	1.63 ± 0.1		3-4	Gallbladder	*Mesonauta festivus*	Bittencourt et al. (2022)

L: spore length; T: spore thickness; T:L thickness/length ratio; CL: capsule length; CW: capsule width; θ: posterior angle; Bob: coils.

* The number of turns in the filament was not identified.

**Figure 3:**
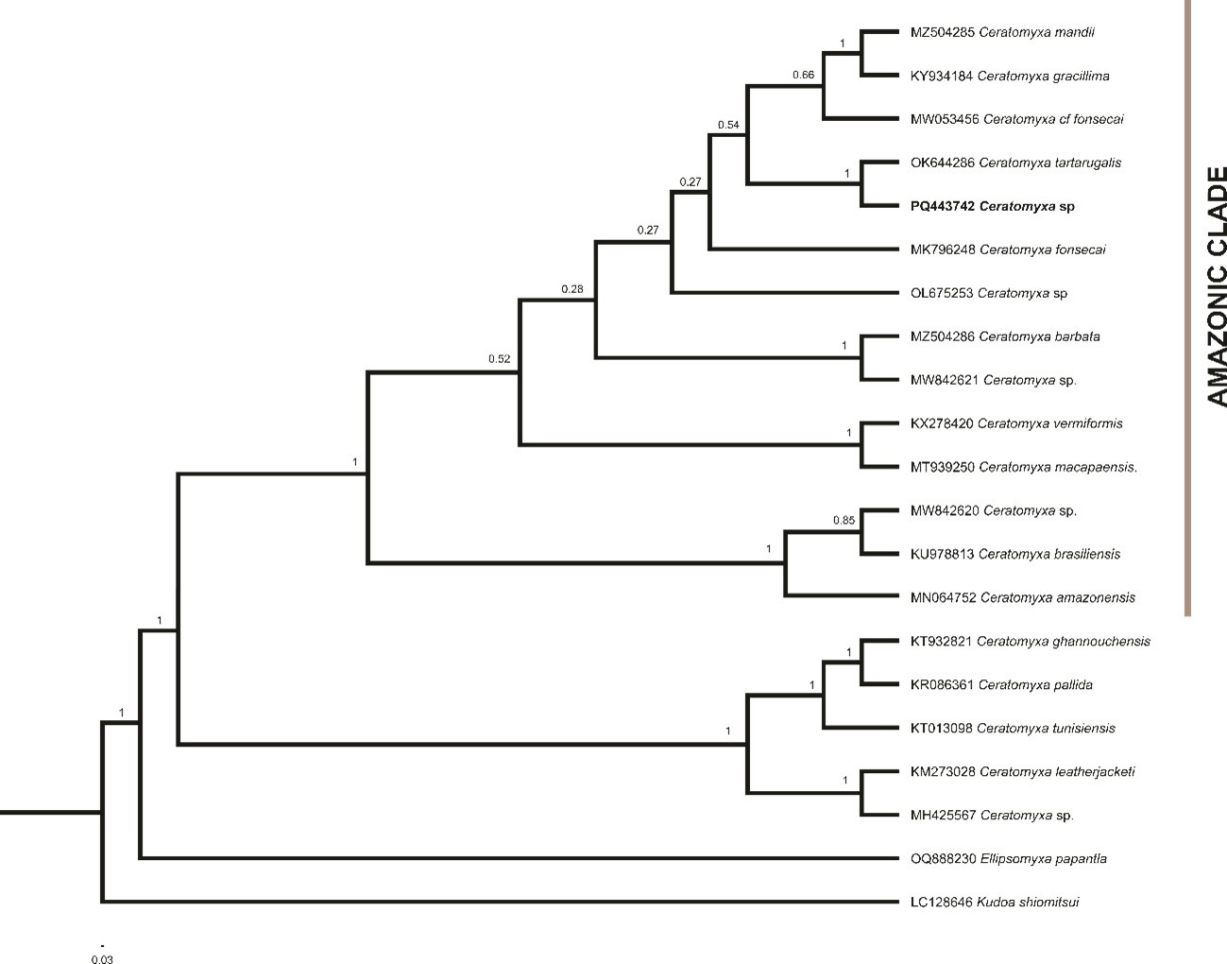
Bayesian phylogenetic tree of the SSU rDNA region, showing the relationships among the *Ceratomyxa* species analysed.

The partial sequence of the SSU rDNA gene for of *Ceratomyxa* sp. obtained in this study comprised 1221 base pairs, with a G + C content of among which 44.64%, distributed as in the follow: A = 29.32%, C = 20.07%, G = 24.57% and T = 26.04%. BLASTn analysis revealed did not reveal no any other sequence with over more than 90% similarity to *Ceratomyxa* sp. The phylogenetic analysis, based on Bayesian inferences, included partial SSU rDNA sequences of other ceratomyxid species that presented more than 75% similarity to the sequence of the present study, as well as additional myxozoan sequences (Figure 3).

The analyses included 19 *Ceratomyxa* species and two additional of other myxozoan species, showing that *Ceratomyxa* sp. belongs to a ceratomyxid clade that includes a the subclade of parasites infecting of Brazilian freshwater fish, primarly mostly from the Brazilian Amazon region, such as *C. tartarugalis*, *C. fonsecai* and *C. vermiformis*. The phylogenetic support confirms the identification of a new *Ceratomyxa* species, with a 15-16% dissimilarity compared to other Brazilian ceratomyxids in the freshwater clade (Figure 3).

Although the observed spore morphology could potentially resemble myxosporids of the genus *Meglitschia*, the absence of unequal capsular foramina lack of molecular data for *Meglitschia* support the inclusion of this myxosporid within the genus *Ceratomyxa*.

Further greater depth of studies, incorporating with ultrastructural analyses and an expanded genetic database, may eventually elucidate relationship between *Ceratomyxa* e *Meglitschia,* as well as their affiliations with other genera within belonging the family Ceratomyxidae.

## References

[B001] Adriano EA, Okamura B (2017). Motility, morphology and phylogeny of the plasmodial worm, *Ceratomyxa vermiformis* n. sp. (Cnidaria: Myxozoa: Myxosporea). Parasitology.

[B002] Araújo PG (2021). Microparasitos eucariotos de Pristobrycon striolatus (Steindachner, 1908) e Hemiodus gracilis (Günther, 1864) oriundos do rio Tartarugalzinho, Amazônia Oriental.

[B003] Araújo BL, Adriano EA, Franzolin GN, Zatti SA, Naldoni J (2022). A novel *Ceratomyxa* species (Myxozoa: Cnidaria) infecting an Amazonian catfish. Parasitol Int.

[B004] Azevedo C, Ribeiro M, Clemente SC, Casal G, Lopes L, Matos P (2011). Light and ultrastructural description of *Meglitschia mylei* n. sp. (Myxozoa) from *Myleus rubripinnis* (Teleostei: Serrasalmidae)in the Amazon River System. J Eukaryot Microbiol.

[B005] Azevedo C, Rocha S, Casal G, São Clemente SC, Matos P, Al-Quraishy S (2013). Ultrastructural description of *Ceratomyxa microlepis* sp. nov (Phylum Myxozoa): a parasite infecting the gall bladder of Hemiodusmicrolepis, a freshwater teleost from the Amazon river. Mem Inst Oswaldo Cruz.

[B006] Barta JR, Martin DS, Liberator PA, Dashkevicz M, Anderson JW, Feighner SD (1997). Phylogenetic relationships among eight *Eimeria* species infecting domestic fowl inferred using complete small subunit ribosomal DNA sequences. J Parasitol.

[B007] Bittencourt LS, Silva DT, Hamoy I, de Carvalho AA, da Silva MF, Videira M (2022). Morphological and phylogenetic features of *Ceratomyxa macapaensis* n. sp. (Myxozoa: Ceratomyxidae) in *Mesonauta festivus* Heckel, 1840 (Cichliformes: Cichlidae) from the Eastern Amazon Region. Acta Parasitol.

[B008] Bush AO, Lafferty KD, Lotz JM, Shostak AW (1997). Parasitology meets ecology on its own terms: Margolis et al. revisited. J Parasitol.

[B009] Diamant A, Whipps CM, Kent ML (2004). A new species of *Sphaeromyxa* (Myxosporea: Sphaeromyxina: Sphaeromyxidae) in devil firefish, *Pterois miles* (Scorpaenidae), from the northern Red Sea: morphology, ultrastructure, and phylogeny. J Parasitol.

[B010] Drummond AJ, Rambaut A (2007). BEAST: Bayesian Evolutionary Analysis by Sampling Trees. BMC Evol Biol.

[B011] Gunter NL, Whipps CM, Adlard RD (2009). *Ceratomyxa* (Myxozoa: Bivalvulida): robust taxon or genus of convenience?. Int J Parasitol.

[B012] Hall TA (1999). BioEdit: a user-friendly biological sequence alignment editor and analysis program for Windows 95/98/NT. Nucleic Acids Symp.

[B013] Heiniger H, Gunter NL, Adlard RD (2008). Relationships between four novel ceratomyxid parasites from the gall bladders of labrid fishes from Heron Island, Queensland, Australia. Parasitol Int.

[B014] Holzer AS, Wootten R, Sommerville C (2007). The secondary structure of the unusually long 18S ribosomal RNA of the myxozoan *Sphaerospora truttae* and structural evolutionary trends in the Myxozoa. Int J Parasitol.

[B015] Lom J, Arthur JR (1989). A guideline for the preparation of species descriptions in Myxosporea. J Fish Dis.

[B016] Lom J, Dyková I (2006). Myxozoan genera: definition and notes on taxonomy, life-cycle terminology and pathogenic species. Folia Parasitol.

[B017] Mathews PD, Naldoni J, Maia AAM, Adriano EA (2016). Morphology and small subunit rDNA-based phylogeny of *Ceratomyxa amazonensis* n. sp. parasite of *Symphysodon discus*, an ornamental freshwater fish from Amazon. Parasitol Res.

[B018] Mooers A, Gascuel O, Stadler T, Li H, Steel M (2012). Branch lengths on birth-death trees and the expected loss of phylogenetic diversity. Syst Biol.

[B019] Okamura B, Gruhl A, Bartholomew JL (2015). Myxozoan evolution, ecology and development..

[B020] Posada D (2008). jModelTest: phylogenetic model averaging. Mol Biol Evol.

[B021] Rambaut A, Drummond AJ, Xie D, Baele G, Suchard MA (2018). Posterior summarisation in Bayesian phylogenetics using Tracer 1.7. Syst Biol.

[B022] Santos GM, Ferreira EJG, Zuanon JAS (2006). Peixes comerciais de Manaus..

[B023] Silva MF, Carvalho AEFB, Hamoy I, Matos ER (2020). Coelozoic parasite of the family Ceratomyxidae (Myxozoa, Bivalvulida) described from motile vermiform plasmodia found in *Hemiodus unimaculatus* Bloch, 1794. Parasitol Res.

[B024] Soares MGM, Costa EL, Siqueira-Souza FK, Anjos HDB, Yakamoto KC, Freitas CEC (2011). Peixes de lagos do médio rio Solimões..

[B025] Swofford DL (2003). PAUP: Phylogenetic Analysis Using Parsimony (and othermethods). Version 4.10b..

[B026] Thabet A, Mansour L, Al Omar SY, Tlig‐Zouari S (2016). *Ceratomyxa tunisiensis* n. sp. (Myxosporea: Bivalvulida) from the gallbladders of two carangid fish caught off the coast of Tunisia. J Eukaryot Microbiol.

[B027] Whipps CM, Adlard RD, Bryant MS, Lester RJG, Findlav V, Kent ML (2003). First report of three *Kudoa* species from eastern Australia: *kudoa thyrsites* from Mahi mahi (*Coryphaena hippurus*), *Kudoa amamiensis* and *Kudoa minithyrsites* n. sp. from sweeper (*Pempheris ypsilychnus*). J Eukaryot Microbiol.

[B028] Zatti SA, Atkinson SD, Maia AAM, Bartholomew JL, Adriano EA (2017). *Ceratomyxa gracillima* n. sp. (Cnidaria: Myxosporea) provides evidence of panmixia and ceratomyxid radiation in the Amazon basin. Parasitology.

